# Dark Tetrad and workplace deviance: Investigating the moderating role of organizational justice perceptions

**DOI:** 10.3389/fpsyg.2022.968283

**Published:** 2022-10-20

**Authors:** Elena Fernández-del-Río, Ángel Castro, Pedro J. Ramos-Villagrasa

**Affiliations:** ^1^Department of Psychology and Sociology, Faculty of Work and Social Sciences, Universidad de Zaragoza, Zaragoza, Spain; ^2^Department of Psychology and Sociology, Faculty of Social and Human Sciences, Universidad de Zaragoza, Teruel, Spain

**Keywords:** Dark Tetrad, dark personality, workplace deviance, counterproductive work behaviors, organizational justice

## Abstract

This study tested the direct effects of Dark Tetrad traits on organizational and interpersonal counterproductive work behaviors (CWBs). We also examined the moderating effects of the three dimensions of organizational justice – distributive justice, procedural justice, and interactional justice – on the Dark Tetrad-CWBs relationships. Based on the data from 613 employees across different occupations, the results revealed that only psychopathy and sadism had significant effects on CWBs targeted at the organization. The results also supported the direct effect of sadism on interpersonal CWBs. The findings confirmed the moderating role of interactional justice but differentially, depending on the dark trait and the target of workplace deviance. Whereas low and medium levels of interactional justice moderated the relationship between Machiavellianism and CWBs directed to the organization, it did not play any role in narcissism, psychopathy, and sadism. Regarding CWBs aimed at other people, interactional justice emerged as a significant moderator in Machiavellianism and sadism. But, whereas sadistic employees performed more harmful behaviors toward other individuals whatever their level of interactional justice, if people high in Machiavellianism (Machs) perceived a high fair interpersonal treatment, they did not show deviant behaviors directed at other employees. The paper concludes with some suggestions and recommendations about the relevance of organizational justice in the influence of dark personality traits on CWBs.

## Introduction

Counterproductive work behaviors (CWBs) can be defined as deliberate actions displayed by employees that damage the well-being of the organization or its members (Sackett and DeVore, 2001). According to several researchers (Robinson and Bennett, 1995; Berry et al., 2007), these behaviors have been clustered in deviant actions targeting individuals (CWBI) and those targeting the organization (CWBO). Considering their personal, economic, and organizational consequences (e.g., Robinson, 2008), there have been strong efforts focused on their prevention, seeking to determine the individual (e.g., dark personality traits) and situational (e.g., perceived organizational justice) antecedents of harmful behaviors at work.

Focusing on dark personality traits, to date, Dark Triad has attracted most the scholars’ research. Instead, research on the relation between the Dark Tetrad on facets of CWBs is still in its infancy (Li et al., 2020). To overcome this gap, the primary aim of this study was to test the direct effects of Dark Tetrad traits, assessed by a workplace-specific measure, on CWBO and CWBI (Wu and LeBreton, 2011; Cohen, 2016). Attending to their idiosyncrasy, it is plausible to consider the existence of differential relationships between the dark traits and the target of workplace deviance. On the other hand, some voices claimed that some relevant mediators and moderators should be considered in the relationships between dark personality traits and CWBs (Cohen, 2016). As Baloch et al. (2017) and Mahmood et al. (2021) pointed out, we should consider that those linkages may be more indirect through other organizational factors than direct. The evidence about the effect of organizational justice in the relationship between dark personality and CWBs is limited to the study by Ying and Cohen (2018). However, these authors focused on Dark Triad, so the role of sadism remains unexplored. Building on these backgrounds, the secondary aim of this paper was to examine the moderating role of perceived organizational justice from a multidimensional perspective.

## Theoretical background and hypothesis development

### Dark Tetrad and counterproductive work behaviors

A great part of the research about individual factors that can lead to CWBs has focused on personality-based variables (e.g., Sackett and DeVore, 2001; Berry et al., 2007; Kish-Gephart et al., 2010). Due to their common dark nature, the associations between Dark Triad traits (i.e., psychopathy, narcissism, and Machiavellianism) and workplace deviance have been verified (e.g., Cohen, 2016; LeBreton et al., 2018; Ying and Cohen, 2018). However, some issues have little empirical evidence or have not even been explored. For instance, literature about the role of everyday sadism, included as the fourth component into the Dark Tetrad (Paulhus, 2014; Međedović and Petrović, 2015), in organizations remains sparse (Gebben et al., 2021; Fernández-del-Río et al., 2021b). Although dark personality traits seem to have a common core, that is callousness (i.e., the lack of empathy toward others; Jones and Figueredo, 2013), several authors defend the existence of particularities that would explain their exhibit markedly different behavior. For instance, Paulhus and Jones (2015) highlighted the grandiose sense of self-importance and entitlement in narcissistic individuals, the planning, coalition-formation, and reputation building typical in Machiavellian people, and the impulsivity and lack of guilty of psychopaths. O’Boyle et al., (2012) meta-analytic review found that all these Dark Triad traits (Paulhus and Williams, 2002) were positively related to CWBs. Instead, as we already mentioned, the relationship between sadism and CWBs is scarce (Li et al., 2020). It seems plausible that the unique traits of sadistic people (e.g.,the enjoyment of cruelty, the subjugating nature; Buckels et al., 2013), would explain that other workers, not organizations, were the targets of their dysfunctional work behavior (e.g., stealing the property of a co-worker or verbally abusing a co-worker). In addition, the cruelty towards other co-workers of people high in everyday sadism would not emerge as a justification of their perception of unfairness at work, but because of the pleasurable nature of these behaviors. Thus, we have expected that Dark Tetrad traits will be significantly related to CWBs but considering the target of deviant actions:

*Hypothesis 1a*: Narcissism, Machiavellianism, and psychopathy will be positively related to CWBO and CWBI.

*Hypothesis 1b*: Sadism will be positively related to CWBI.

Additionally, previous research has used general measures instead of specific measures of dark personality traits in a workplace setting, although some voices defend their utility and pertinency (Woo et al., 2015; Thibault and Kelloway, 2020). Therefore, the predictive validity of a contextual measure of the Dark Tetrad over workplace deviant behaviors needs more evidence.

### Organizational justice

Situational antecedents must also be considered in the prediction of attitudes and behaviors at work (e.g., Sangperm, 2017). Specifically, previous research emphasizes the central role of perceived organizational justice in the CWBs domain (e.g., Cohen-Charash and Spector, 2001; Hershcovis et al., 2007; Cohen, 2016). In fact, Sackett and DeVore (2001) affirmed that “there is a certain poetry in behaving badly in response to some perceived injustice” (p. 160).

Based on the equity perception (Adams, 1965), the concept of organizational justice has evolved over time from a single-factor perspective (i.e., distributive justice) to a multidimensional approach. Distributive justice refers to “the perceived fairness of the outcomes one receives from social exchange or interaction” (Nowakowski and Conlon, 2005, p. 5). Procedural justice is defined as the perceived fairness associated with the procedures which affect the outcome distributions (Leventhal, 1980). Interactional justice concerns the quality of the interpersonal treatment that people receive when procedures are implemented (Bies and Moag, 1986).

The meta-analysis of Colquitt et al. (2001) provided strong evidence that these three dimensions of organizational justice were empirically different from each other. In fact, CWBs have differential relationships with distinct types of organizational justice. Cohen-Charash and Spector (2001) reported similar weighted mean correlations between procedural and distributive justice and CWBs (−0.22, and −0.28, respectively). Also, both procedural and distributive justice were similarly related to conflict with others at work (weighted mean *r* = −0.18 and −0.19, respectively). Unfortunately, they did not examine the relations between interactional justice and CWBs because of the low number of primary studies. Berry et al. (2007) found that interactional and procedural justice had moderate negative correlations with CWBI and CWBO (−0.20 to −0.25), whereas distributive justice showed weaker correlations (−0.07 to −0.17).

According to previous evidence, the study established the following hypothesis:

*Hypothesis 2*: Distributive justice, procedural justice, and interactional justice will be negatively related to CWBO and CWBI.

### Dark personality, CWBs, and organizational justice

As we remarked on, despite this idiosyncrasy, dark personalities share some features that influence their behavioral repertoire in diverse settings, such as in the workplace (Schyns, 2015). For instance, highly narcissistic individuals would be likely to react negatively if they receive negative feedback about their performance (Barry et al., 2006; Campbell et al., 2011). In addition, if their expectancies about the fairness of the distribution of burdens and benefits and the process used to arrive at decisions are not met or they consider they are not treated sensitively and respectfully by authorities and third parties (i.e., threat to their self-esteem and their superiority), these individuals could be more likely to perform CWBs (Penney and Spector, 2002; Grijalva and Harms, 2014; Grijalva and Newman, 2015). The above explanation could be generalized to Machiavellian individuals. As previous research indicates (e.g., Schmitt et al., 2005; Foote and Harmon, 2006) high Machs are prone to be sensitive to justice violations. Within the organizational context, for instance, Machs who perceive that their professional interests, status, and/or career have been damaged or the achievement of their objectives (e.g., being denied in a promotion) has been blocked (Jones and Paulhus, 2009; Zettler et al., 2011) could engage in CWBs. Thus, low perceived organizational justice could serve as a triggering mechanism to perform deviant behaviors against the organization or other employees (e.g., Zheng et al., 2017). In psychopaths, besides their tendency toward impulsive behavior and their lack of remorse (e.g., Hare, 2003), interpersonal manipulation and criminal tendencies are key features of their behavioral repertoire (Mahmut et al., 2011). In this sense, sub-clinical psychopathy may be useful for predicting CWBs, especially interpersonal deviance (Scherer et al., 2013). Those deviant behaviors could be even more frequent under the perception of having been unfairly treated in the workplace. In this line, Schilbach et al. (2020) pointed out that subclinical psychopathy was associated with unfavorable cognitive appraisal tendencies (e.g., increased obstruction and threat appraisal of the workday) which in turn may be a precursor for CWBI and CWBO.

As a consequence of its recent incorporation into the dark personality space, we have no robust evidence about the relationship between sadism and workplace deviant behaviors as well as the influence of situational factors on it. Considering that this trait cannot be reduced to other dark traits (Johnson et al., 2019), we must deepen our understanding of the sadism-CWBs relationship. Due to their key feature (i.e., intrinsic appetitive motivation to inflict suffering on innocent others; Buckels et al., 2013), the cruelty towards other co-workers of people high in everyday sadism would not emerge as a justification for their perception of unfairness at work, but instead the pleasurable nature of such behaviors.

Therefore, we have hypothesized:

*Hypothesis 3*: Distributive justice, procedural justice, and interactional justice will moderate the relationships between Dark Tetrad traits (except sadism) and CWBs.

**Figure 1:**
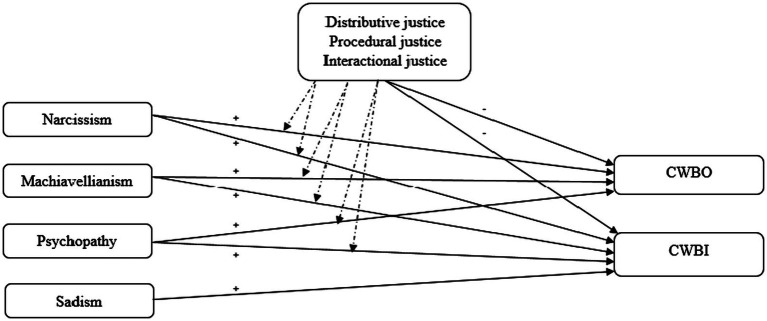
Conceptual model. Note. A continuous arrow indicates a direct relation. A discontinuous arrow indicates a moderating relation. CWBO = counterproductive work behaviors targeting the organization; CWBI = counterproductive work behavious targeting individuals.

Thus, the present paper is focused on deepening into the moderating role of perceived organizational justice in the relationship between Dark Tetrad of personality and the two clusters of workplace deviant behaviors as it is shown in Figure 1.

## Materials and methods

### Participants and procedure

A total of 613 employees (*M* age = 38.78, *SD* = 14.06; 54% women) from different organizations participated in this study. Their average job tenure was 8.38 years (*SD* = 10.09).

Data were collected with a non-probability sampling technique (i.e., convenience sampling). Authors requested their university students to cooperate, distributing the questionnaire to the workers they knew in any kind of job position. Students received training in questionnaire completion to provide the necessary support to their recruits. Workers who voluntarily agreed to participate were informed about the research objectives of this study and the confidentiality and anonymity of their responses. Seven hundred and twenty questionnaires were distributed, and six hundred and twenty-five were returned (86.8%). After removing those with missing values in any variables of interest, statistical analysis was performed with data from six hundred and thirteen employees (85.1%). This sample has been used in previous research (Fernández del Río et al., 2020; Fernández-del-Río et al., 2021b), although other variables, instruments, and research questions were considered.

### Measures

A paper-and-pencil questionnaire was designed to measure sociodemographic and work behavior characteristics, dark personality traits, perceived organizational justice, and counterproductive work behaviors.

#### Sociodemographic and work characteristics

We asked participants about their gender, age, and job tenure.

#### Dark personality

We applied the Spanish version of the Dark Tetrad at Work scale (DTW) by Thibault and Kelloway (2020), which was adapted by Fernández del Río et al. (2020). This scale comprises 22 items rated on a 5-point Likert type scale ranging from 1 = *strongly disagree* to 5 = *strongly agree*. It measures narcissism (e.g., “Others admire me at work”; α = 0.61), Machiavellianism (e.g., “I do not trust others at work”; α = 0.75), psychopathy (e.g., “I’m rather insensitive at work”; α = 0.78), and sadism (e.g., “I would laugh if I saw someone get fired”; α = 0.91).

#### Organizational justice

We applied the scale of Moliner et al. (2008). It is a 12-item instrument rated on a 7-point Likert scale, ranging from 1 = *strongly disagree* to 7 = *strongly agree*. This measure reflects the concepts of distributive (e.g., “The rewards I receive here are quite fair”; α = 0.95), procedural (e.g., “Procedures used in this company to evaluate my work are fair”; α = 0.91), and interactional justice (e.g., “My supervisor offers adequate justification for decisions made about my job”; α = 0.91).

#### Counterproductive work behaviors

The Workplace Deviance Scale (Bennett and Robinson, 2000) contains 19 items assessing how often an individual has engaged in CWBs towards the organization (e.g., “Littered your work environment”; α = 0.83), and towards individuals (e.g., “Cursed at someone at work”; α = 0.85) in the past year rated on a 7-point scale ranging from 1 = *never* to 7 = *daily*. We used those items included in the Spanish version adapted by Fernández del Río et al. (2021a).

### Analytic strategy

We computed descriptive statistics, Pearson and point-biserial correlations, and reliabilities (Cronbach’s α). Hierarchical moderated regressions were conducted to examine the moderating influence of three types of organizational justice in the relationship between each dark personality trait and CWBs. For each dimension of CWBs, all control variables (gender, age, job tenure) were entered in first step, and all four dark traits (narcissism, Machiavellianism, psychopathy, sadism) and the three forms of organizational justice perceptions (distributive, procedural, interactional) were entered in second step of the regression analyses. Twelve two-way interactions comprised of the cross-product of each of the four dark personality traits with each of the three types of organizational justice were entered in the third step. We also contemplated the problem of multicollinearity between predictors considering variance inflation factors (VIF) according to the recommendations of Hair et al. (2010), that is, VIF values must be less than 10.0. Bias-corrected bootstrapping (with 10,000 resamples) was used to generate confidence intervals for the hypotheses tested.

We plotted the moderating effect of perceived organizational justice on CWBs across low, medium, and high levels of Dark Tetrad traits (+1 *SD*; Aiken and West, 1991). All the statistical analyses were performed in SPSS 26 software.

Harman’s single factor test was applied to detect common method bias in this study (Brewer et al., 1970). The total variance for a single factor was 19.75% (< 50%; Fuller et al., 2016), so we concluded that common method bias did not affect the data.

## Results

### Correlations between dark tetrad, organizational justice, and CWBs

Descriptive statistics, reliabilities, and correlations of the study variables are presented in Table 1. Regarding the criteria, except for narcissism, all Dark Tetrad traits correlated positively with CWBO [*M*r = 0.31, range (0.22, 0.36)], and CWBI [*M*r = 0.34, range (0.19, 0.45)]. According to Cohen's (1992) criterion for effect size (i.e., 0.10–0.29 is small, 0.30–0.49 is medium, 0.50 or higher is large), those relations were ranged from small to medium.

**Table 1:** Descriptive statistics, reliabilities, and bivariate relations of the variables.

	Descriptives			Associations										
	*M*	*SD*	α	1	2	3	4	5	6	7	8	9	10	11
**Pearson Correlations**
1. Gender	0.46	0.50												
2. Age	38.78	14.06		0.09*										
3. Job tenure (years)	8.38	10.09		0.11**	0.64***									
4. Narcissism	17.47	3.16	0.61	0.14**	0.09*	0.07								
5. Machia-vellianism	10.84	3.30	0.75	0.03	−0.07	−0.09*	0.02							
6. Psychopathy	10.34	3.46	0.78	0.21***	−0.01	0.02	0.14**	0.35***						
7. Sadism	8.20	3.44	0.91	0.12**	−0.03	−0.01	0.21***	0.28***	0.67***					
8. Distributive justice	16.94	6.79	0.95	0.02	−0.01	0.01	0.17***	−0.19***	−0.05	−0.04				
9. Procedural justice	17.27	6.52	0.91	0.03	−0.06	−0.03	0.22***	−0.24***	−0.09*	−0.04	0.76***			
10. Interactional justice	21.10	6.21	0.91	0.02	−0.08	−0.07	0.15***	−0.27***	−0.17***	−0.15***	0.36***	0.57***		
11. CWBO	18.87	7.96	0.83	0.09*	−0.18***	−0.17***	−0.01	0.22***	0.36***	0.34***	−0.13**	−0.19***	−0.19***	
12. CWBI	9.44	4.75	0.85	0.14***	−0.12**	−0.09*	0.07	0.19***	0.37***	0.45***	−0.12**	−0.15***	−0.19***	0.68***

*N* = 613. Gender: 0 = women, 1 = men; CWBO: counterproductive work behaviors targeting the organization; CWBI: counterproductive work behaviors targeting individuals.

* *p* < 0.05;

** *p* < 0.01;

*** *p* < 0.001.

Associations between dark personality traits and the potential moderating variables were also significant but not in the same direction. Whereas narcissism correlated positively with all subtypes of organizational justice [*M*|r| = 0.18, range (0.15, 0.22)], Machiavellianism showed negative correlations [*M|*r| = −0.23, range (−0.27, −0.19)]. Psychopathy presented significant but small correlations with procedural (*r* = −0.09) and interactional justice (*r* = −0.17), and sadism was only significantly correlated with interactional justice (*r* = −0.15).

Both types of CWBs showed negative, albeit small, correlations with all three dimensions of organizational justice [*M*|r| = −0.17, range (−0.19, −0.13) for CWBO; *M*|r| = −0.15, range (−0.19, −0.12) for CWBI].

### Moderation role of three types of perceived organizational justice

Hierarchical moderated regressions were conducted to examine the moderating role of three types of organizational justice in the relationship between Dark Tetrad and CWBs. Table 2 shows the first and second step of the regression analyses for each dimension of CWBs. The interaction terms in the third step were reported in Table 3. For all analyses the VIF scores were lower than 10.0, which suggest that there were no problems with multicollinearity.

**Table 2:** Results for hierarchical regression analyses for CWBO and CWBI.

	CWBO			CWBI		
	*β*	*t*	VIF	*β*	*t*	VIF
**Step 1**						
Gender	0.10	2.45*	1.01	0.15	3.59***	1.01
Age	−0.15	−2.85**	1.72	−0.15	2.72**	1.70
Job tenure	−0.08	−1.52	1.73	−0.01	−0.20	1.71
Adjusted *R*^2^	0.05			0.04		
*F*	10.27***			8.11***		
**Step 2**						
Gender	0.05	1.31	1.07	0.10	2.59*	1.07
Age	−0.12	−2.41*	1.75	−0.11	−2.33***	1.73
Job tenure	−0.11	−2.12*	1.75	−0.04	−0.81	1.73
Narcissism	−0.03	−0.65	1.15	0.02	0.51	1.15
Machiavellianism	0.04	0.98	1.24	−0.01	−0.21	1.25
Psychopathy	0.17	3.23**	1.96	0.07	1.27	1.96
Sadism	0.22	4.24***	1.88	0.37	7.51***	1.88
Distributive OJ	0.03	0.58	2.39	−0.01	−0.13	2.42
Procedural OJ	−0.17	−2.57*	3.13	−0.10	−1.59	3.16
Interactional OJ	−0.03	−0.67	1.61	−0.09	−1.93	1.61
Adjusted *R*^2^	0.21			0.25		
∆*R*^2^	0.17			0.22		
*F*	15.98***			19.61***		
∆F	17.53***			23.57***		

*N* = 613. CWBO: counterproductive work behaviors targeting the organization; CWBI: counterproductive work behaviors targeting individuals. OJ: Organizational Justice.

* *p* < 0.05;

** *p* < 0.01;

*** *p* < 0.001.

**Table 3:** Hierarchical moderated regression analysis for three types of organizational justice as moderators in the relationships between dark personality traits and CWBs.

	CWBO			CWBI		
	*β*	*t*	VIF	*β*	*t*	VIF
**Step 3**						
Gender	0.06	1.50	1.10	0.10	2.65**	1.10
Age	−0.13	−2.72**	1.79	−0.11	−2.35**	1.77
Job tenure	−0.09	−1.86	1.80	−0.03	−0.65	1.78
Narcissism	−0.03	−0.63	1.18	0.02	0.47	1.18
Machiavellianism	0.05	1.08	1.31	−0.01	−0.06	1.31
Psychopathy	0.17	3.16**	2.04	0.08	1.57	2.04
Sadism	0.21	3.91***	2.03	0.34	6.71***	2.03
Distributive OJ	0.04	0.65	2.60	−0.03	−0.57	2.63
Procedural OJ	−0.18	−2.77**	3.22	−0.09	−1.46	3.25
Interactional OJ	0.01	0.14	1.70	−0.05	−1.13	1.70
N × DOJ	−0.03	−0.51	2.75	0.02	0.28	2.79
N × POJ	0.08	1.24	3.37	0.01	0.05	3.42
N × IOJ	0.01	0.14	1.70	0.02	0.38	1.71
M × DOJ	0.01	0.07	3.11	0.04	0.55	3.21
M × POJ	0.03	0.37	3.88	0.01	0.05	3.99
M × IOJ	−0.14	−2.84**	1.79	−0.11	−2.29*	1.80
P × DOJ	0.08	0.97	5.36	−0.06	−0.71	5.38
P × POJ	−0.08	−0.77	7.31	−0.03	−0.27	7.32
P × IOJ	−0.02	−0.31	3.52	0.11	1.59	3.52
S × DOJ	−0.01	−0.16	5.73	0.02	0.19	5.80
S × POJ	0.05	0.49	8.10	0.05	0.41	8.17
S × IOJ	−0.09	−1.25	3.92	−0.21	−2.89**	3.92
Adjusted *R*^2^	0.23			0.26		
∆*R*^2^	0.04			0.03		
*F*	8.67***			10.23***		
∆F	2.23*			2.05*		

*N* = 613. CWBO: counterproductive work behaviors targeting the organization; CWBI: counterproductive work behaviors targeting individuals. N: narcissism; M: Machiavellianism; P: psychopathy; S: sadism; DOJ: distributive organizational justice; POJ: procedural organizational justice; IOJ: interactional organizacional justice.

*p < 0.05; **p < 0.01; ***p < 0.001.

**Figure 2:**
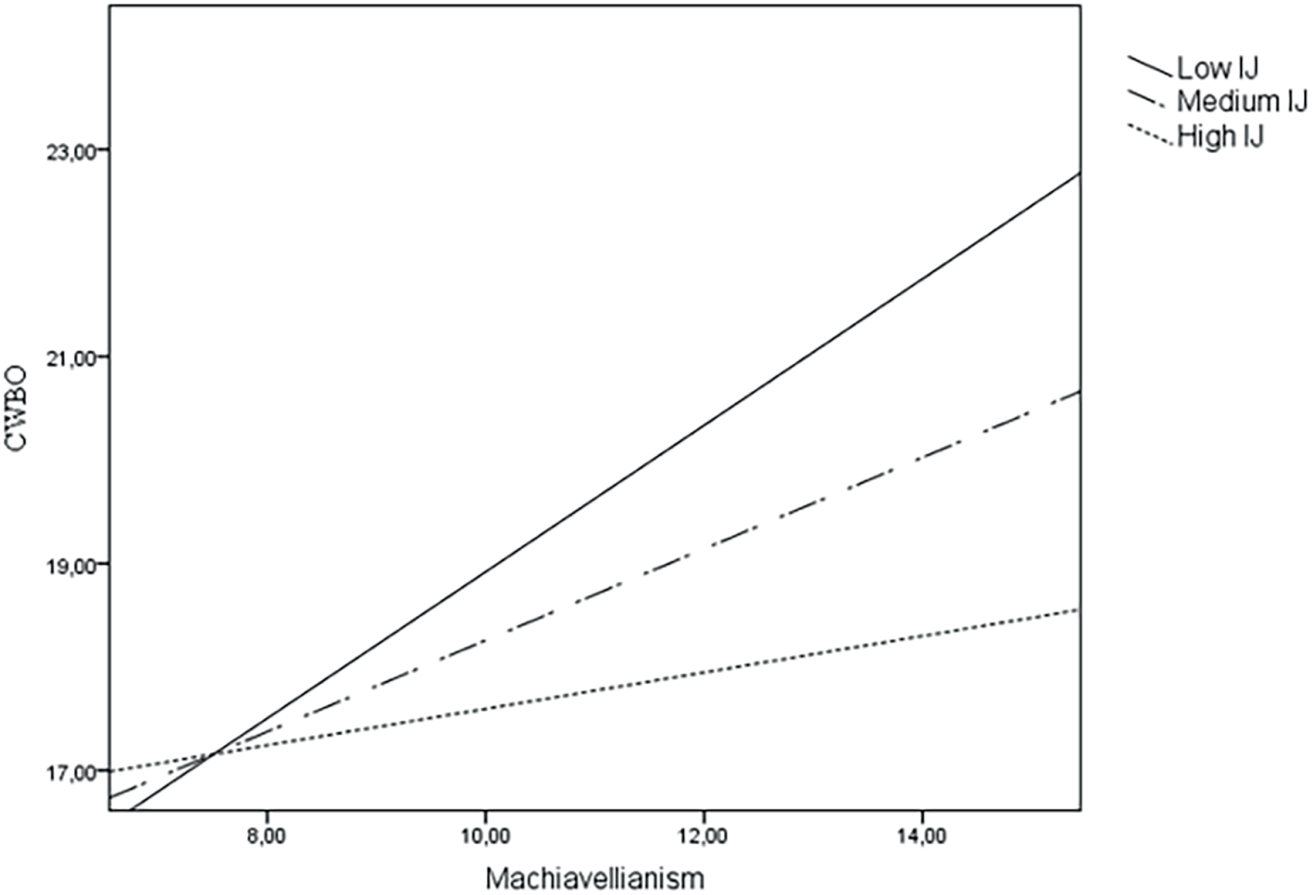
Moderating effects of perceived interactional justice (IJ) on the relationship between Machiavellianism and counterproductive woek behaviors targeting the organization (CWBO).

Regarding CWBO, all the increments in R^2^ were statistically significant for all the steps (*p* < 0.001 for Step 2; *p* < 0.05 for Step 3). The incorporation of the Dark Tetrad and all the three types of organizational justice added 17% of explained variance (Table 2), mainly due to a positive contribution of psychopathy and sadism (positive sign), and procedural justice (negative sign). As Table 3 shows, there was only one case of moderation: perceived interactional justice significantly moderated the effects of Machiavellianism on CWBO. Based on the interaction plot shown on Figure 2, under low (*B* = 0.71, *p* < 0.001) or medium (*B* = 0.44, *p* < 0.001) levels of interactional justice, the effect of high scores on Machiavellianism on deliberate actions that harm the organization was stronger. That is, there seems to be no relationship between Machiavellianism and CWBO if employees perceived a high quality of their interpersonal treatment (*B* = 0.18, *p* = 0.21).

**Figure 3:**
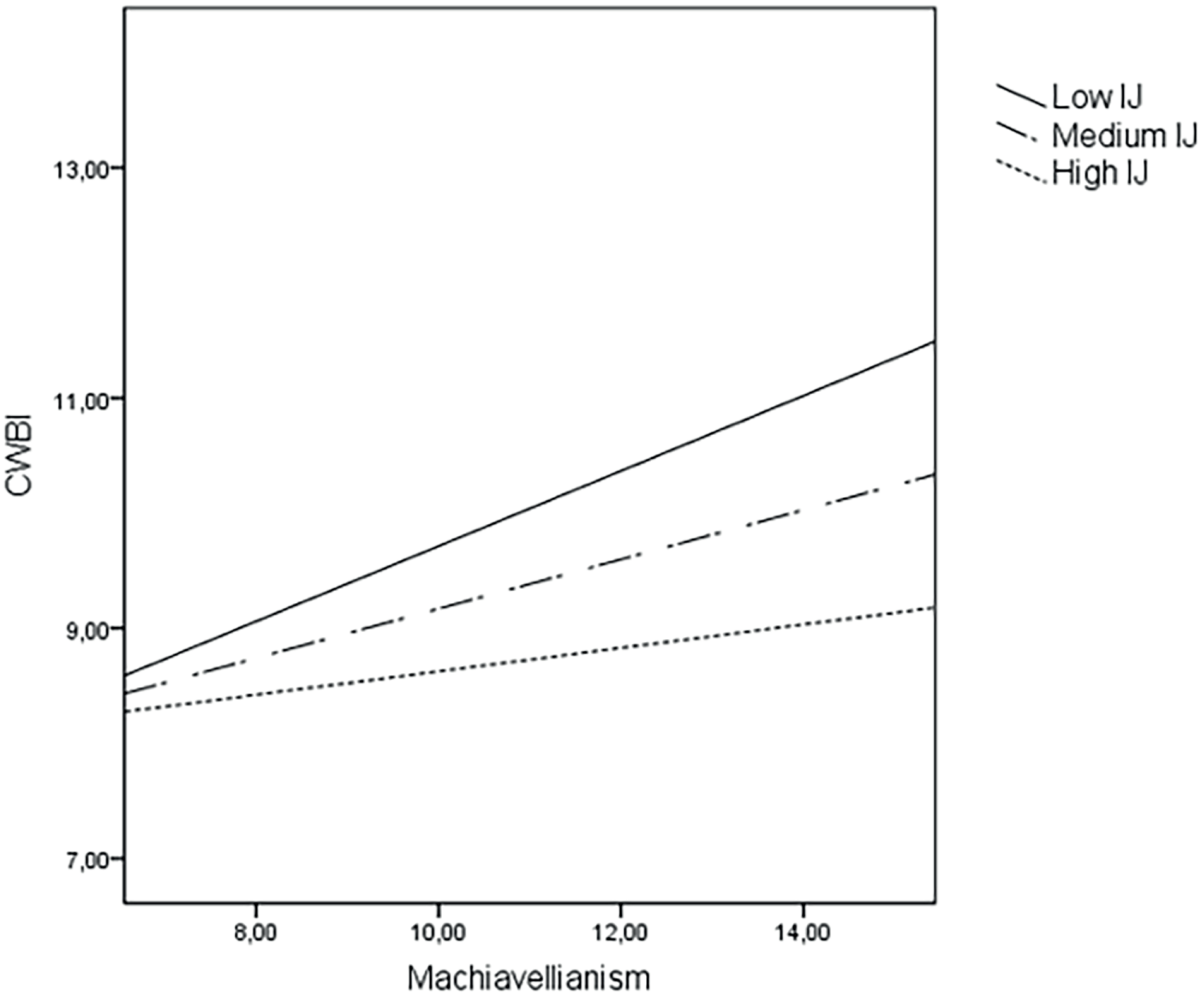
Moderating effects of perceived interactional justice (IJ) on the relationship between Machiavellianism and counterproductive work behavious targeting indiviauals (CWBI).

Concerning CWBI, findings revealed that the inclusion of the Dark Tetrad and all the three types of organizational justice added 22% of explained variance (Table 2), mainly due to mainly due to a positive contribution of sadism. As shown in Table 3, perceived interactional justice moderated the relationship between Machiavellianism and CWBI. Figure 3 shows that the positive association between Machiavellianism and CWBI was stronger when the perception of interactional justice was low (*B* = 0.33, *p* < 0.001) or medium (*B* = 0.21, *p* < 0.001). However, if Machs perceived that they were treated with politeness, dignity, and respect by authorities or third parties, there seems to be no relationship between this dark trait and CWBI (*B* = 0.10, *p* = 0.22).

**Figure 4:**
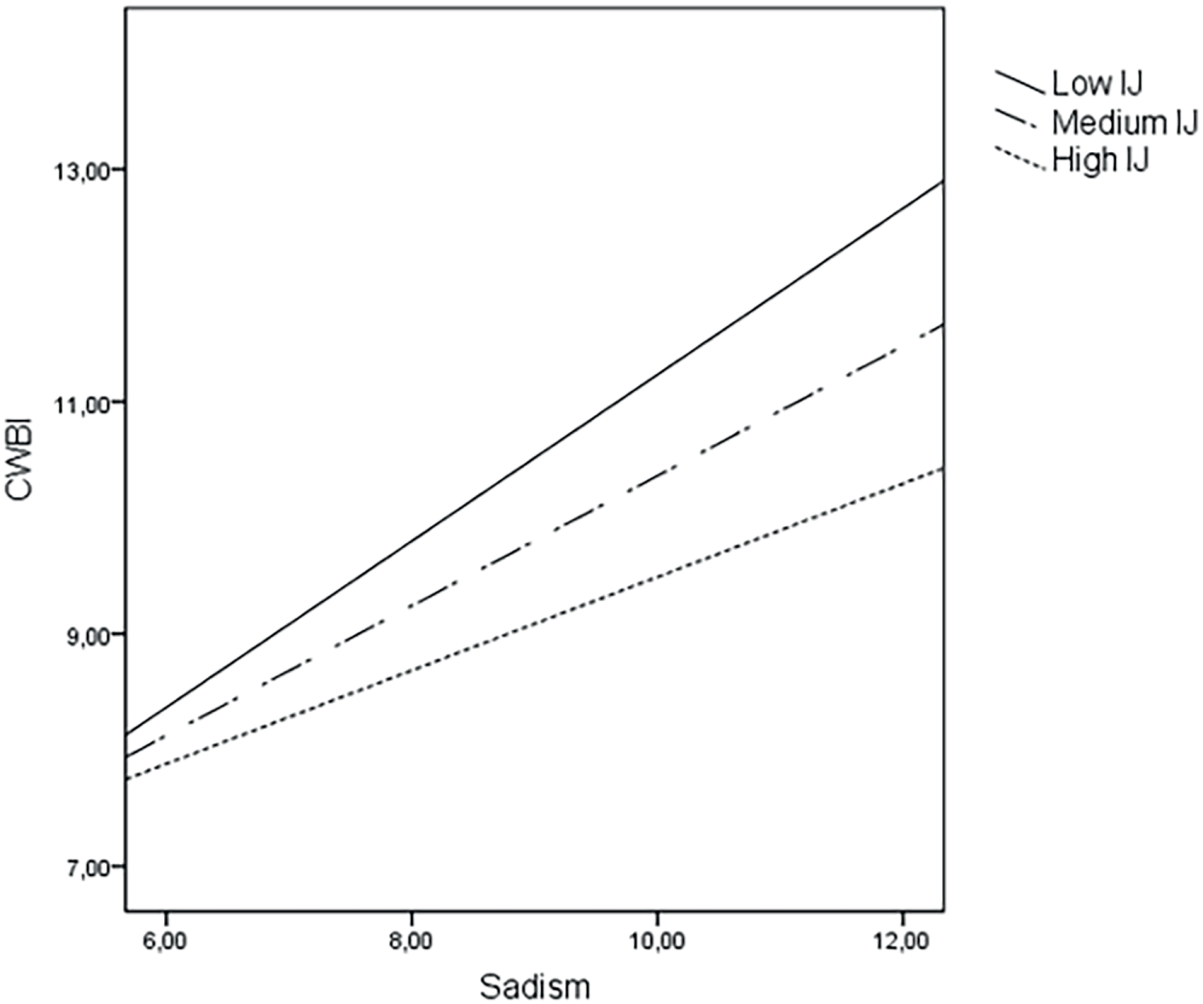
Moderating effects of perceived interactional justice (IJ) on the relationship between sadism and counterproductive work behavious targeting indiviauals (CWBI).

There is also support for significant moderation in the case of sadism (Figure 4): sadistic employees performed more harmful behaviors toward other individuals whatever their level of interactional justice (*B* = 0.72, *p* < 0.001, for low level; *B* = 0.56, *p* < 0.001, for medium level; *B* = 0.40, *p* < 0.001, for high level), although the effect was weaker when the perceived justice is higher.

## Discussion

Although prior research suggested that narcissism, Machiavellianism, and psychopathy are associated with CWBs (O’Boyle et al., 2012), there was no evidence about the role of everyday sadism proposed as an addition to the Dark Triad, making a Dark Tetrad. We make a novel contribution by examining these relationships based on the target of CWBs (organizational vs. interpersonal). In addition, we provide evidence about the moderating role of organizational justice from a multidimensional approach. Overall, our findings suggest that some dark personality traits may be effective predictors of CWBs especially under difficult or trying conditions related to the perception of equity in the relationship between supervisors and employees. Only the dimension of organizational justice concerned in terms of encounters, not just exchanges (i.e., interactional justice) moderates Dark Tetrad-CWBs relationships.

Concerning H1a, results indicate that employees who possess high Machiavellianism, psychopathy, or sadism tend to report higher levels of CWBO and CWBI. This is consistent with previous literature (O’Boyle et al., 2012; Ying and Cohen, 2018; Li et al., 2020), which points out that individuals high in any dark personality trait tend to engage in a variety of deliberate actions that harm the organization or its members. Psychopathy provided a modest contribution to predicting CWBO compared to sadism (β =0.17 and β = 0.22 respectively), and it did not emerge as a significant predictor when the criterion variable was CWBI, contrary to previous evidence that contemplates the target of deviant behaviors (e.g., Schilbach et al., 2020). According to Thibault and Kelloway (2020), who designed the measure of Dark Tetrad used in the present study, this could be explained by the overlapping variance of psychopathy with sadism. In addition, no one type of organizational justice played a moderating role in the psychopathy-CWBO relationship. Joining Thibault and Kelloway, we call for future research to examine the effects of subclinical psychopathy, assessed with a contextualized measure, on workplace deviant behaviors.

As regards H1b, although sadism was also positively related to CWBO, as Thibault and Kelloway (2020) found, the association with CWBI was the strongest among dark traits, and higher than in the case of CWBO. In fact, hierarchical regression analysis revealed that sadism was the only significant predictor of CWBI. As Buckels et al. (2013) pointed out, sadists possess an “intrinsic appetitive motivation to inflict suffering on victims” (p. 2207), a motivation that is not present in other dark personalities. They are referred to as cruel aggressors that impose physical and psychological pain on others for their pleasure and enjoyment (Chester et al., 2019). Therefore, it seems logical to expect that this key feature of sadists will also be manifested in organizational settings. In fact, Fernández-del-Río et al. (2021b) found that sadism was the strongest personality predictor of bullying others at work, improving the explained variance over the other dark traits.

Contrary to our hypothesis, narcissism was not directly related to both types of CWBs. One possible explanation for such a finding could be the content of items for narcissism included in the scale that has been used (i.e., Dark Tetrad at Work Scale). They are mainly focused on the grandiose/exhibitionism and the leadership/authority facets of narcissism, not on the entitlement/exploitativeness dimension. According to previous evidence, the grandiose/exhibitionism dimension was unrelated to CWBs (Grijalva and Newman, 2015). Besides, if the low reliability of this scale is considered, our findings should be interpreted cautiously.

Regarding demographics, we also want to stress that the findings are consistent with prior workplace studies and meta-analyses. Males and young employees were more likely to score high on CWBs, especially those targeting individuals (Berry et al., 2007; Ng et al., 2016; Fernández del Río et al., 2021a,b).

In our study, we also found that all-four dark traits were associated with organizational justice, but in different ways. Narcissism was slightly correlated, in a positive sense, to organizational justice. This could be related to the specificity of the measure (i.e., developed in a workplace setting; Thibault and Kelloway, 2020) or some characteristics of this dark trait, such as an inflated sense of self and a sense of superiority that could be influencing their perceptions. That is, the recognition of unfairness could be socially interpreted as a sign of weakness, so narcissistic employees avoid presenting themselves as vulnerable or weak workers in front of others or showing a lack of dominance and superiority (Paulhus and Williams, 2002). On the contrary, Machiavellianism was negatively correlated to all three types of organizational justice, especially interactional justice. One of the defining features of Machs, i.e., a cynical worldview (Jones and Paulhus, 2009), could be influencing their justice perceptions in organizational contexts. Machiavellianism is also associated with low agreeableness in the five-factor model of personality, and this trait is an important correlate of organizational justice (Shi et al., 2009). In the case of psychopathy and sadism, both dark traits showed negative, but weak, associations with interactional justice. Some common characteristics shared by psychopaths and sadists, as the lack of empathy and readiness for emotional involvement (Kirsch and Becker, 2007), inflicting suffering on others, could explain their negative associations with organizational justice focused on interpersonal treatment.

Regarding H2, results revealed that all types of organizational justice have a direct, negative, and significant relationship with both types of CWB consistent with previous findings (Cohen-Charash and Spector, 2001). As Berry et al. (2007) suggested, distributive justice showed weaker correlations compared to procedural and interactional justice, especially in the case of CWBI. This would support the interdependence of three types of organizational justice (Greenberg and Colquitt, 2005).

Our moderating analyses (H3) indicated that perceived interactional unfairness tends to act specifically as a situational antecedent but just in the case of two dark personality traits: Machiavellianism and sadism. Employees with higher levels of Machiavellianism who reported low or medium levels of interactional justice engaged more frequently in CWBO and CWBI compared to their Machs counterparts with high interactional justice. If Machiavellian employees considered that their supervisors treat them without enough respect and dignity (e.g., not listening to their concerns, not providing adequate explanations for decisions, demonstrating a lack of empathy for the other person’s plight) were more likely to perform deviant behaviors against their organization (e.g., neglecting to follow their boss’s instructions) and/or their co-workers (e.g., acting rudely toward someone at work). Instead, the perception of being treated with social sensitivity may have an inhibiting effect on CWBs.

Regarding sadism, all levels of interactional justice moderated the relationship with interpersonal deviant behaviors, not with CWBO. This suggests that sadists performed CWBI more frequently, whatever the degree of perceived interactional injustice was, maybe due to the “pleasurable” nature of inflicting pain to other coworkers. But, it is worth noting that these employees reported increased frequency of their interpersonal deviance when the perceived treatment by supervisors worsened. A promising future direction could include exploring the links between sadism and types of aggression (hostile vs. instrumental) at work (Bushman and Anderson, 2001). According to our findings, hostile aggression (i.e., causing harm for its own sake) would be more likely than instrumental aggression (i.e., using harm as a means to some other end) in sadist employees. But, the influence of cognitive predictors (e.g., perceptions of organizational injustice) should also be considered because of its strong relationship with instrumental aggression (Bowling and Gruys, 2010).

### Limitations and future directions

The current study has a number of limitations that deserve mention here. First, the use of convenience sampling could have affected the representativeness of the sample and the generalization of the results. In addition, the cross-sectional design prevents the inference of causality. Second, the scale used to assess the Dark Tetrad is a tailored-made and job-context personality measure, so comparisons with past research are limited. According to some authors, the use of more contextualized personality measures improves the prediction of performance, at least in academic settings (Holtrop et al., 2014), so future studies carried out in organizational contexts should introduce contextual measures of dark personality. Third, the effect sizes of moderation analysis were modest, so findings should be interpreted cautiously. The study was somewhat narrow in scope as it focused on only one situational variable as a moderating variable in the Dark Tetrad-CWBs relationships. Furthermore, the two dimensions of interactional justice (informational and interpersonal) defended by Greenberg (1993) and Bies (2005) should be considered in the analysis of the association between Dark Tetrad and interactional justice in the prediction of several facets of job performance.

Further research should seek to examine the mediating/moderating role of other individual differences (e.g., affective predictors, moral beliefs). For instance, a promising future question research is whether moral disengagement could be a mediator of the relationship between the dark personality and CWBs, in line with the recent work by Erzi (2020). As the study of Dark Tetrad in organizational settings is still in its youth, we recommend continuing research on the workplace deviant behaviors, especially to better understand the underlying motivations and strategies of each dark personality pattern when the individual misbehaves at the workplace.

### Conclusion

The present study makes a significant contribution by going deeper into the examination of the role of the relationship between the Dark Tetrad and CWBs and the influence of situational factors reported by O’Boyle et al. (2012). We also attended to the comment of Cohen (2016) on the pertinence of exploring the links between diverse dark constructs considering the dimensions of CWB differentially and the proposal of using workplace-specific measures of the dark personality (Thibault and Kelloway, 2020). In line with previous studies, we appreciate that perceived organizational justice must also be considered in the prediction of job performance (e.g., Wu et al., 2016; Kerdpitak and Jermsittiparsert, 2020). Concretely, we conclude that interactional justice represents an important situational factor that may enhance the expression of CWBO and CWBI among Machiavellian employees. Regarding practical implications, managers should invest time and resources to create an environment that dissuades people from such harmful activities through, for instance, improving the interpersonal treatment they receive at the hands of organizational decision-makers (e.g., treating individuals with dignity, and providing subordinates with justifications or explanations). These efforts should be accompanied by full transparency about why procedures are used in a certain way or why outcomes are distributed in a particular fashion. This seems to be essential in Machs employees because of their propensity to the untrustworthy view of human nature (Jones and Paulhus, 2009). We also should pay attention to the presence of sadistic traits on employees, especially if they occupy a job position of authority over other people. Their intrinsic tendency to experience pleasure from other people’s physical or psychological suffering could affect subordinate’s well-being and job-related attitudes in the same way of corporate psychopathy (e.g., Mathieu et al., 2014).

## Data availability statement

The raw data supporting the conclusions of this article will be made available by the authors, without undue reservation.

## Ethics statement

Ethical review and approval was not required for the study on human participants in accordance with the local legislation and institutional requirements. The patients/participants provided their written informed consent to participate in this study.

## Author contributions

EF-d-R, ÁC, and PR-V contributed conception, design of the study, and wrote the second draft of the manuscript. EF-d-R and PJ-V organized the database and performed the statistical analysis. EF-d-R wrote the first draft of the manuscript. All authors contributed to the article and approved the submitted version.

## Funding

This work was supported by Government of Aragon (Group S31_20D). Department of Innovation, Research and University and FEDER 2014–2020, “Building Europe from Aragón.”

## Conflict of interest

The authors declare that the research was conducted in the absence of any commercial or financial relationships that could be construed as a potential conflict of interest.

## Publisher’s note

All claims expressed in this article are solely those of the authors and do not necessarily represent those of their affiliated organizations, or those of the publisher, the editors and the reviewers. Any product that may be evaluated in this article, or claim that may be made by its manufacturer, is not guaranteed or endorsed by the publisher.

## Supplementary material

The Supplementary material for this article can be found online at: https://www.frontiersin.org/articles/10.3389/fpsyg.2022.968283/full#supplementary-material
