# Supplementary material for: Dark Tetrad and workplace deviance: Investigating the moderating role of organizational justice perceptions
Source: Front Psychol. 2022 Oct 20;13:968283. doi: 10.3389/fpsyg.2022.968283 (PMC9631426; doi:10.3389/fpsyg.2022.968283)
Supplement: Supplementary file 1 [file Data_Sheet_1.pdf]

Supplementary Material

1.1 Spanish translation of the Dark Tetrad at Work scale (DTW; Thibault & Kelloway, 2020) by Fernández-del-Río et al. (2020)

Por favor, indique su grado de acuerdo o desacuerdo con cada una de las siguientes afirmaciones conforme a la siguiente escala:

1 = Totalmente en desacuerdo
2 = En desacuerdo
3 = Ni de acuerdo ni en desacuerdo
4 = De acuerdo
5 = Totalmente de acuerdo

1. Mi puesto en el trabajo es prestigioso.
2. Soy mucho más valioso que mis compañeros de trabajo.
3. Exijo respeto en el trabajo.
4. La gente siempre me presta atención en el trabajo.
5. Los demás me admiran en el trabajo.
6. Me gusta ser el centro de atención en el trabajo.
7. No confío en los demás en el trabajo.
8. En el trabajo siempre tienes que mirar por tu propio interés.
9. En el trabajo la gente se "apuñala" con tal de salir adelante.
10. En el trabajo la gente solo está motivada por las ganancias personales.
11. No me importa si mi comportamiento en el trabajo perjudica a los demás.
12. Me han dicho que actúo precipitadamente en el trabajo.
13. Cuando estoy en el trabajo, no suelo pensar en las consecuencias de mis actos.
14. Me gusta aprovecharme de mis compañeros de trabajo.
15. Soy bastante insensible en el trabajo.
16. No me importa si perjudico accidentalmente a alguien en el trabajo.
17. Me encanta ver a mi jefe gritándole a mis compañeros de trabajo.
18. Puedo dominar a otros en el trabajo usando el miedo.
19. Es divertido ver a la gente cometer errores en el trabajo.
20. Nunca me canso de burlarme de mis compañeros de trabajo.
21. Me reiría si viese que despidieran a alguien.
22. Fantaseo sobre hacer daño a gente con la que trabajo.

1.2 Spanish translation of the Workplace Deviance Scale (WDS; Bennett & Robinson, 2000) by Fernández-del-Río et al. (2021)

A continuación, encontrará una serie de comportamientos. Por favor, para cada uno de ellos indique, de acuerdo con la siguiente escala, con qué frecuencia los ha realizado en el último año:
1= Nunca
2 = Una vez al año
3 = Dos veces al año
4 = Varias veces al año
5 = Mensualmente
6 = Semanalmente
7 = A diario

1. Llevarse algún bien de la empresa sin permiso.
2. Pasar demasiado tiempo fantaseando o soñando despierto en lugar de trabajar.
3. Burlarse de alguien en el trabajo.
4. Falsificar un recibo para que le devuelvan más dinero del que gastó en algún asunto de la empresa.
5. Decir algo hiriente a alguien en el trabajo.
6. Hacer una pausa más larga o más pausas de las que son aceptables en su trabajo.
7. Hacer una broma de tipo étnico, religioso o racial en el trabajo.
8. Llegar tarde al trabajo sin permiso.
9. Tener desordenado su lugar de trabajo.
10. Insultar a alguien en el trabajo.
11. Desobedecer las instrucciones de su jefe.
12. Trabajar intencionalmente más despacio de lo que podría hacerlo.
13. Hablar de información confidencial de la empresa con una persona no autorizada.
14. Gastar una broma pesada a alguien en el trabajo.
15. Ser grosero con alguien en el trabajo.
16. Consumir drogas o alcohol en el trabajo.
17. Esforzarse poco en su trabajo.
18. Avergonzar públicamente a alguien en el trabajo.
19. Dejar trabajo pendiente para conseguir horas extra.
